# mtDNAcombine: tools to combine sequences from multiple studies

**DOI:** 10.1186/s12859-021-04048-0

**Published:** 2021-03-09

**Authors:** Eleanor F. Miller, Andrea Manica

**Affiliations:** Department of Zoology, University of Cambridge, Downing Street, Cambridge, CB2 3EJ UK

**Keywords:** Demographic history, R package, Mitochondrial DNA, Public datasets, Bayesian skyline plots

## Abstract

**Background:**

Today an unprecedented amount of genetic sequence data is stored in publicly available repositories. For decades now, mitochondrial DNA (mtDNA) has been the workhorse of genetic studies, and as a result, there is a large volume of mtDNA data available in these repositories for a wide range of species. Indeed, whilst whole genome sequencing is an exciting prospect for the future, for most non-model organisms’ classical markers such as mtDNA remain widely used. By compiling existing data from multiple original studies, it is possible to build powerful new datasets capable of exploring many questions in ecology, evolution and conservation biology. One key question that these data can help inform is what happened in a species’ demographic past. However, compiling data in this manner is not trivial, there are many complexities associated with data extraction, data quality and data handling.

**Results:**

Here we present the mtDNAcombine package, a collection of tools developed to manage some of the major decisions associated with handling multi-study sequence data with a particular focus on preparing sequence data for Bayesian skyline plot demographic reconstructions.

**Conclusions:**

There is now more genetic information available than ever before and large meta-data sets offer great opportunities to explore new and exciting avenues of research. However, compiling multi-study datasets still remains a technically challenging prospect. The mtDNAcombine package provides a pipeline to streamline the process of downloading, curating, and analysing sequence data, guiding the process of compiling data sets from the online database GenBank.

## Background

Understanding a species’ demographic past can help inform many questions in ecology, evolution and conservation biology. Consequently, there is a lot of interest in methods that are able to infer how a population’s size may have changed through time. Traditional methods relied on insight from the fossil record [1–3]. However, although fossils are informative about many species, including our own, they remain a limited resource with coarse geographic and temporal resolution. In contrast, genetic methods have the potential to offer better resolution and are now established as the primary means by which a population’s past can be interrogated.

**Fig. 1:**
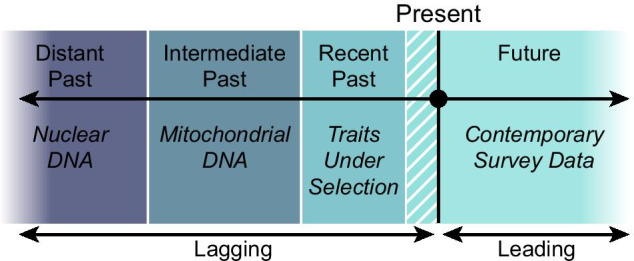
Utility of different loci for reconstructing different periods of population history. Adapted from Zink and Barrowclough [7]

In brief, characteristics of genetic data act as an archive of past population dynamics and can provide insight into historical demographic events. Whilst there is a wide array of genetic markers for studies to choose from, different markers have different attributes. Levels of sensitivity and temporal resolution vary between each class of marker, with different markers often reflecting different aspects of a population’s biology. Highly variable regions of an individual’s genome, such as mitochondrial DNA (mtDNA), can be informative for the recent past (Fig. 1) and are, therefore, important markers for reconstructing demographic changes through time.

MtDNA has been used widely for demographic reconstruction. The haploid nature of mtDNA along with its rapid rate evolution [4], lack of recombination [5] and uniparental mode of inheritance [6] make it more sensitive to capture changes in population size than slower evolving nuclear genes [7] (Fig. 1). MtDNA therefore has the temporal resolution to capture the impacts of relatively recent events that might be of interest, such as the Last Glacial Maximum (LGM). In combination with coalescent-based reconstruction methods such as Bayesian Skyline Plots (BSPs) [8], mtDNA can be used to estimate a detailed population profile that stretches back tens, or even hundreds, of thousands of years.

Aside from mtDNA, other highly variable regions of the genome exist, such as certain regions of the Y chromosome and microsatellite sites. The Y chromosome can be a valuable tool when investigating population demographics through time, offering insight into the male-specific history of a species e.g. [9]. However, due to its highly repetitive nature, and the risk of co-amplification of homologous X-chromosome regions, typing the Y chromosome is challenging and this marker has classically been overlooked in genetic studies [10, 11]. As a result, there is less publicly available data for this biomarker than for the more dominant mtDNA marker.

Microsatellites can also be highly informative in reconstructing past demography, and have been used extensively in non-model systems [12]. However, besides the expense of developing microsatellites for any given system (primarily due to the cost of primer development) [13], a major limitation is the difficulty of merging data from multiple sources [14, 15]. Because of difficulty in consistently typing the number of repeats, merging can only be accomplished when a subset of samples or at least populations have been typed in multiple studies to help calibration [14, 15].

With the falling costs of whole genome sequencing (WGS) and the growing interest in large scale sequencing projects, such as the Bird 10,000 Genomes Project (B10K) [16], the availability of WGS data is rapidly increasing. Using a single, high quality, diploid genome sequence, the pairwise sequentially Markovian coalescent (PSMC) method [17] can reconstruct a profile of population size through time for that species. However, PSMC is limited in its ability to capture details of population history more recently than ~ 1000 generations ago [18]. The multiple sequential Markovian coalescent (MSMC), a method that builds on the PSMC framework, somewhat resolves this issue, using data from multiple individuals to improve the resolution of PSMC by an order of magnitude to more recent times [18]. However, this method is costly, requiring multiple, phased, high-quality genomes from the species of interest. Whilst phasing data may get easier as average sequenced read lengths increases, this is still a non-trivial step and phased data is frequently too difficult or costly to obtain for non-model species.

Whilst WGS is an exciting prospect for the future, for most non-model organisms’ classical markers such as mtDNA remain widely used [19]. Indeed, the falling costs of high throughput DNA sequencing, coupled with routine deposition of project data into public databases such as the National Centre for Biotechnology Information’s (NCBI) GenBank [20], has created a burgeoning resource of mtDNA sequence data. For the first time, these databases contain sufficient sequence data to allow users to build quality meta-datasets. Although individual studies may only be able to undertake spatially and temporally restricted sampling efforts, by creatively using pre-existing resources from multiple studies, it is now feasible to improve sampling strategy, range coverage and sample sizes without additional sampling. As the workhorse of population genetics studies for many decades, public domain mtDNA data are available in large numbers for a wide range of species across most higher taxa.

**Fig. 2:**
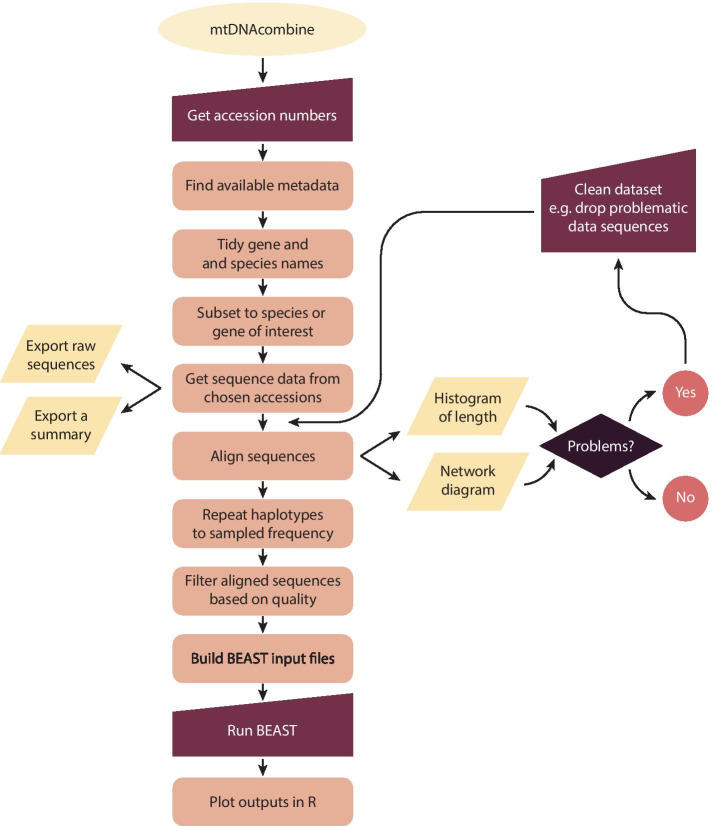
Flow diagram of mtDNAcombine pipeline showing decisions and steps supported by the package

Although sequence databases are normally curated, data input is generally not standardised or error checked. Studies differ greatly in the length and identity of target sequence, the quality of sequence curation and, while some studies upload all sequences obtained, others merely upload unique haplotypes. There are also instances of incorrect sample assignation. Altogether, this means that to compile a comparable set of sequences from multiple studies requires extensive data processing. In the current paper, we consider the practicalities and problems faced by a meta-analysis of publicly available data and present the mtDNAcombine package. The mtDNAcombine package is a collection of tools developed to manage some of the major decisions associated with handling multi-study sequence data with a particular focus on preparing sequence data for BSP population demographic reconstructions (Fig. 2.).

## Implementation

Here we will illustrate the utility of mtDNAcombine in an investigation into how the effective population sizes (*N*_e_) of a number of Holarctic avian species have changed since the last deglaciation, a typical application of BSPs. We chose to investigate five species that have been widely sequenced; the white wagtail (*Motacilla alba*), the Eurasian three-toed woodpecker (*Picoides tridactylus*), the purple sandpiper (*Calidris maritimaI*), the common rosefinch (*Carpodacus erythrinus*), and the pine grosbeak (*Pinicola enucleator*); searching for NADH dehydrogenase subunit 2 (ND2) sequence data as it is one of the most frequently uploaded avian mitochondrial genes in GenBank.

A more technical and implementation focused walk-through can be found on mtDNAcombine’s GitHub landing page as a package vignette.

### Raw data

To begin a genetic study of any species using publicly available DNA from multiple individuals, and studies, it is first necessary to locate the sequence data that will be used. The most common way to find these data is to search an annotated DNA database such as GenBank, which is the main public repository for nucleotide sequence data, for the species of interest.

Initially we ran a search for any mtDNA sequence data for each of our five species in GenBank, downloading the list of unique accession numbers each search retrieved. This produced an initial list of 525 accessions, stored as a csv file. mtDNAcombine was then used to import meta information (e.g. species full scientific name, length of the available sequence) about relevant accessions into a data frame. Whilst scraping information, mtDNA combine will also handle the potential duplication due to the presence of NCBI Reference Sequence (RefSeq) project [21] accessions. RefSeq aims to curate records and associated data, providing a set of reference standards for studies from all disciplines to draw on. As these data are drawn from the International Nucleotide Sequence Database Collaboration [INSDC, which consists of GenBank, the European Nucleotide Archive (ENA), and the DNA Data Bank of Japan (DDBJ)] databases, a basic search can recover two accessions for the same sample; the RefSeq accession and the source record(s). In this instance, the duplicates can be distinguished because all RefSeq records include an underscore (“_”) in their accession number, while simple repository accessions never have this character. mtDNAcombine silently identifies and removes these accessions, having first checked that the 'original' accessions they duplicate are already included in the dataset. This can lead to a reduced number of unique accession numbers retained compared to the number originally searched. In the data for our five species there were two RefSeq sequences that were excluded in this way, one for the white wagtail and one for the pine grosbeak, bringing the number of unique accessions down to 523.

Note that each accession number can be associated with multiple pieces of information. Some of these are simply redundant coding of the same element: within GenBank, the same single sequence is often associated to multiple “feature” tags (e.g. 'source', 'gene', and 'CDS'). However, this practice is inconsistent, and in some cases different abbreviations for a given gene are used under different “feature” tags. An additional complication is that every submission to GenBank receives a unique accession number but these individual submissions can contain data for anything from a single gene through to whole genome data which includes a large number of genes, each of which is a separate feature tag in the database. In mtDNAcombine, we start by simply scraping all information tags and generating additional items for each of them (stored as rows in a dataframe); these will be later processed to be reduced to a set of coherent unique items. In our example, an initial scrape of the 523 unique accessions generated an initial tally of 1240 items.

It should be noted that, although GenBank staff review all submissions to GenBank, and quality control checks are performed before release, there is no standardised format for entering descriptive information. As a result, features such as alternative abbreviations for gene names, deprecated species names, subspecies names, and simple misspellings are all common. When nomenclature does not match between entries filtering a large database for comparable samples becomes complex and it is therefore important for mtDNAcombine to support standardisation of sequence metadata.

Looking at our species data in more detail it is clear that the different studies that had submitted data for the white wagtail had included different levels of detail for taxonomic rank in the ‘organism’ field, with some entries including subspecies names. In this instance we want to group these data together under the larger species umbrella so, using the mtDNAcombine function ‘standardise_spp_names’, we revised the subspecies names back to species names.

As individual studies upload data to GenBank using a range of different synonyms, abbreviations, and misspellings, it was also important to control for the breadth of possible names used to describe a single gene. Therefore, we standardised nomenclature across the data frame using the ‘standardise_gene_names’ function. By default this function loads a file containing alternative abbreviations, common misspellings, and other frequent errors, for 18 commonly sequenced mtDNA genes, converting matching gene names to a given set of standard nomenclatures. If mitochondrial DNA is not the focus of a study, users can upload a custom conversion list by specifying a new input file when using the function.

To retain only information on the specific mitochondrial gene needed for our BSP analysis (ND2), we further filtered the data frame with the ‘gene_of_interest’ function. This function allows the user to both drop any additional genes associated with accessions that may hold more than one gene, and/or drop any accessions that do not contain the gene of interest, depending on how the original accession list was created. In our study, ND2 had been part of the original search term so every unique accession included ND2. Therefore, this step brings the number of observations in our data frame back to 523 (after removing the RefSeq duplications). mtDNAcombine then used this data frame of curated accession metadata to download relevant raw sequence data for our study species.

### Data processing

After sequence data were obtained, they needed to be aligned in order to capture comparable regions of the genome. A number of public domain software programs are available that can achieve this, including T-Coffee [22], MUSCLE [23, 24] and MAFFT [25]. However, in order to keep the pipeline as simple and automated as possible, mtDNAcombine uses ClustalW [26], implemented through the R package ‘msa’ [27]. Though BEAST can handle missing or ambiguous bases [28], we consider it best to use alignments without gaps or ambiguities. Whilst some insertions or deletions may be genuine, when working with sequences from multiple sources, the data are likely to have been sequenced with different techniques to varying standards. Inclusion of basic sequencing errors could affect later analyses, and the volume or type of errors will not be consistent across all studies, nor across all taxa. Therefore, to ensure consistent sequence quality, mtDNAcombine has the ability to remove all sites with ambiguities, insertions, deletions, and missing data.

For any group of studies, there will be numerous reasons the samples were original collected and sequenced. Each project will have had, among other things, a different budget, time constraints, target area of the mitochondrial genome, and available sequencing technology, meaning that different lengths of the genome/target gene will have been sequenced. In some instances, some studies will only have sequenced a very short section of the gene of interest. If the number of base pairs (bp) is too low, the sample is unlikely to hold enough information to be informative for population demographic reconstruction. As a result, the mtDNAcombine is designed to drop individual accessions that are below a user-set threshold before processing the data. There can be no out-of-the-box value for this ‘minimal length’ as the most appropriate size will vary with a wide range of factors such as the gene under investigation, mutation rate, absolute gene length, and the available sample size. However, as a baseline we set the minimal sequence length (‘minbp’ parameter) to 200 bp at this stage, excluding any samples that clearly hold insufficient information for our downstream analysis before moving onto aligning and cropping sequences. In our five species, this step did not result in the exclusion of any samples as all sequences were over 300 bp long.

Another issue to consider is the amount of overlap among sequences from different studies. Automatically cropping all the sequences to the maximum overlap length may result in the loss of a large amount of data unbeknownst to the user. Therefore, to make the process of alignment and sequence trimming transparent, one of the diagnostic plots produced by mtDNAcombine is a histogram showing the original variation in sequence length as well as the length of the trimmed, maximum overlap, dataset (more details on the diagnostic plots produced by mtDNAcombine can be found in the ‘Diagnostic plots’ section in the vignette). This plot flags instances where a large number of base pairs have been removed in order to include a shorter sequence.

**Fig. 3:**
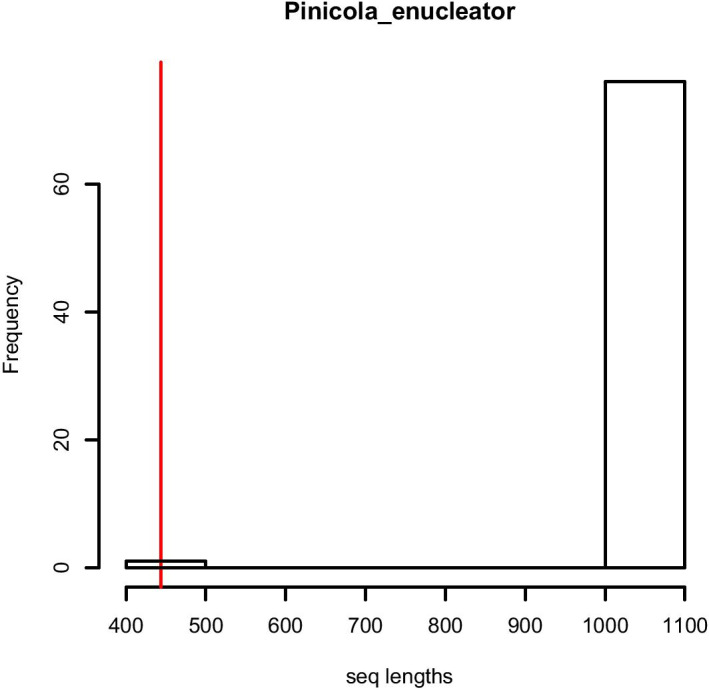
Diagnostic histogram plot for sequence trimming in the ‘align_and_summarise’ function. The maximum overlapping length (red line) shown on a histogram of sequence lengths before processing

Sequence length versus sample size is a trade-off that the user will want to weight differently depending on the data available. mtDNAcombine allows the user to inspect the results of trimming, go back, revise the strategy by removing certain samples, and repeat the process. For example, we found that, in the pine grosbeak dataset, the majority of sequences had been heavily cropped (losing > 500 bp) due to the inclusion of one shorter sequence (Fig. 3). In this instance we decided to return to the original input files and remove the raw sequence data for that one, short sample before returning to the pipeline with the edited set of samples.

Aside from the region, length, and quality of data, studies also differ in the ways they deposit data. Some upload a single copy of each haplotype found, while others upload sequences for each individual sampled. As haplotype frequency can drive estimates of *N*_e_ and alter the reconstructed timings of demographic events, datasets built exclusively of unique haplotypes are not suitable for BSP analysis [29]. It was therefore important we confirmed if any studies included in our dataset had uploaded only unique haplotypes. Where this happens, it is vital to find the number of samples these haplotypes represent, or data from the study must be excluded. However, routinely checking every source publication to confirm the repository deposition style is a non-trivial task and may become impractical for larger analyses. Therefore, to guide this process, the mtDNAcombine ‘align_and_summarise’ function automatically flags studies where all samples are unique haplotypes (i.e. there are no replicates) as candidates for further investigation.

For three of the species in our data set (the white wagtail, the purple sandpiper, and the common rosefinch) mtDNAcombine flagged papers that needed further review. Reading the original published studies showed that, for the white wagtail and common rosefinch*,* the studies had in fact created a new accession for every individual sampled and no action was required. Small sample sizes in these studies meant every sample was unique, triggering the requirement for manual inspection. However, only representative haplotype sequences had been uploaded for the purple sandpiper, rather than a new GenBank accession being created for every sample. Therefore, we manually extracted original sampled frequency information from the literature and used the ‘magnify_to_sampled_freq’ function to update our dataset with this information. This was a critical step as, for example, one haplotype, represented by a single GenBank accession had been sampled 50 times. See ‘MAGNIFY_Calidris_maritima.csv’ file in mtDNAcombine package extdata folder for further details.

Another feature of genetic data known to cause problems for demographic reconstructions methods, including BSP analysis, is population sub-structure [30–33]. BSP analysis, like other coalescent methods, is founded on the Wright–Fisher model and hence assumes panmixia [34]. This assumption is violated by population sub-structure [30, 35], which acts to reduce the probability that lineages from different demes coalesce. In practice, depending on the sampling strategy employed, sub-structure can lead to inflated population size estimate in older parts of the reconstructed history but can also noticeably reduce apparent population size at the present [30]. Accurate demographic reconstruction therefore requires careful consideration of whether sub-structure is or might be present.

Depending on the level of supplementary detail available for each sample, the decision to split a population for analysis can be simple. For example, in instances where sampling location data are available and clear geographic divisions coincide with major genetic clades, datasets can be separated and multiple sequence files handled as individual datasets. However, it is important not to over-split the data. Clades are a natural feature even of fully homogeneous populations, so if any obvious clades are removed, what is left will tend to be star-like haplotype clusters. Such clusters will often yield a signal of population expansion which may or may not be real. Deciding if and where to divide datasets remains one of the more subjective and difficult challenges and it can be worth investing time into running data sub-sets to determine the impact of alternative splitting decisions. To aid these decision, the mtDNAcombine ‘align_and_summarise’ function automatically draws haplotype network diagrams within R using the package ‘pegas’ [36]. For our species of interest, inspection of the haplotype networks did not reveal any extreme clustering, and so the data were left unaltered for all five species.

Another data quality concern would be the presence of extreme outliers: haplotypes represented by a single sample that were separated from all others by many base changes. Such outliers may be genuine but equally may reflect immigrant individuals, sample mislabelling [37], amplification of integrated nuclear copies, incorrect accession codes, or even result from poor-quality sequencing. We feel that the benefits of including these outliers in case they are genuine are far outweighed by the risk that they distort the process of inference. Therefore, within the ‘outliers_dropped’ function, any samples identified as “extreme outliers” are removed from the working dataset, with a new fasta file that excludes the highly diverged sample being automatically written out for use in downstream analysis. We recognise that a multitude of factors (e.g. life history, population history, data availability, data quality) will influence the criteria for data inclusion. Therefore, the degree of separation from other haplotypes necessary for a sample to be classified as an “extreme outlier” can be defined by the user. We set this value to > 30 base pairs for each of our five species, leading to the removal of one sample from the white wagtail population (Additional file 1: Fig. S1). No other sample was found to be that divergent, and all other datasets remained unchanged.

At this stage, each dataset had been fully processed with all available sequences having been through the mtDNAcombine processing pipeline. The sample sizes ranged between 17 and 208 individuals and sequence lengths between 332 and 1038 bp. Before progressing to the next phase of analysis, we needed to do undertake a final quality control measure. For BSP analyses to effectively capture past population dynamics, the input data needs to contain sufficient information. With an analysis based on a single mtDNA gene, we felt that species with less than 6 haplotypes, less than 20 samples, or less than 600 bp would be unlikely to provide enough information to BEAST to reliably capture patterns of population’s past demography and therefore excluded these datasets. This was easily done by sequentially running the ‘drop_low_sample_size’, ‘drop_low_haplo_number’, and ‘drop_low sequence_length’ functions. We therefore dropped the three-toed woodpecker (only 5 haplotypes) and the pine grosbeak (sequences of only 444 bp) datasets, leaving three species that we considered likely to provide robust BSP analysis (Table 1.).

**Table 1.** Details of datasets built from GenBank after processing in mtDNAcombine pipeline

Species name	No. of haplotypes	Seq. length	Sample size
*Picoides tridactylus*	5	332	30
*Pinicola enucleator*	37	444	77
*Calidris maritima*	12	744	73
*Carpodacus erythrinus*	115	1038	190
*Motacilla alba*	57	906	209

### Setting up and running BEAST

#### BEAST input

The mtDNAcombine package function ‘setup_basic_xml’ utilises the ‘babette’ package [38] to build basic XML files from the data set processed earlier in the pipeline. This tool is key, particularly for large comparative studies, where as many steps as possible should be kept constant. Minimising the number of manual steps needed both speeds up the process and reduces the opportunity for the introduction of human error, as well as making outputs as directly comparable as possible. We used the ‘setup_basic_xml’ function to create comparable skeleton XML files for each of our three species data sets, editing specific parameters (e.g. mutation rate, model choice, output names) to be data set specific.

One significant parameter choice for BSP analysis is that of the mutation rate, yet selection of an appropriate mutation rate is a persistent problem in genetic studies. Both the mutation rate itself and its associated confidence will vary between taxa, and it is necessary to consider how best to standardise this to maximise consistency across profiles when running a large-scale comparative study. For birds, recent work [39] proposes that body-mass can be used to inform more accurate calculations of taxon-specific substitution rates and provides a correction factor for variation in rates according to body mass as well as major mtDNA loci. We therefore created dataset-specific mutation rates using body mass/gene correction factors from Nabholz et al. [39] ‘Calibration set 4’ (3rd codon position) and body mass data from Dunning et al. [40].

Once parameterisation decisions had been made and the XML input files finalised, we ran our samples in BEAST2, using the BEAGLE library [41] since this can significantly improve the speed of a run.

#### BEAST output

Interpretation of BEAST outputs has been covered well in the literature e.g. [29, 30] and by those who designed and built the software [42–46]. As with any statistical model, checks need to be done to confirm the reliability of the output. In BEAST2 these are generally undertaken using the software package Tracer [47] and focus on appropriate convergence of the Markov chain. As a rule of thumb, outputs should be treated with caution wherever the effective sample sizes (ESS) for a given parameter drops below 200. Whilst ESS values can be captured in R through the package ‘babette’ [38], we think that a visual inspection of each run in Tracer is best practice. Therefore, we reviewed each of the three BSP analyses in Tracer v1.6, confirming that all three of our datasets converged successfully with ESS values > 200, before exporting the extensive summary data.

BSPs can be drawn using Tracer, however, for more flexibility, and to facilitate exploration of the profiles in greater detail, the mtDNAcombine package vignette (section ‘Exploring outputs’) presents example code for plotting BSP profiles in R using the Tracer summary data. As it is anticipated that data presentation will be highly project specific this code is not tied up in functions, enabling easy editing and adaptation by the user. We chose to visualise the three reconstructed profiles from our illustrative dataset in R using this code.

## Results

**Fig. 4:**
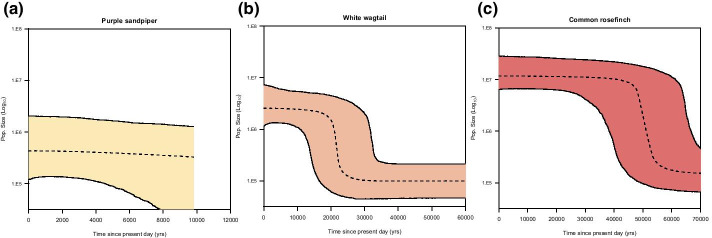
BSP profiles for three species, median *N*_e_ is a dashed line and 95% highest posterior density intervals (HPD) are marked by a coloured region. **a** Purple sandpiper, **b** white wagtail, **c** common rosefinch

The three profiles show different reconstructed demographic profiles for the three species. Firstly, analysis of the purple sandpiper has returned a near flat profile (Fig. 4a). This may indicate a stable population history for the species, however, it may also result from a lack of power to detect an expansion [29]. As sample sizes are not large for this species (73 individuals) we would caution over interpretation of this profile.

The other two profiles indicate that these populations underwent large demographic expansion in the past. The white wagtail profile suggests a rapid increase in population size that initiates around 25–30 kya, with the population size stabilising again around 15–20 kya (Fig. 4b). On the other hand, the reconstructed profile for the common rosefinch indicates a major population expansion around 50–60 kya (Fig. 4c).

## Discussion

Here we present the mtDNAcombine package, providing a pipeline to streamline the process of downloading, curating, and analysing sequence data from GenBank (Fig. 2). With the exponentially expanding volume of data in public DNA sequence repositories, there is now more genetic information available than ever before. Building large meta-data sets by combining existing data offers the opportunity to explore new and exciting avenues of research e.g. [48–50]. However, compiling such datasets remains a technically challenging prospect. Unknown sequence quality, little control over sampling structure, potential errors in species identification, and limited control of sample size, are all factors that can negatively affect a comparative study if not carefully handled.

mtDNAcombine has been specifically designed to tackle the challenges associated with sourcing and aggregating data from multiple studies, with a focus on demographic reconstruction. Whilst other tools exist that tackle sections of this process, such as downloading [51] and aligning [23] sequences, the package we present here offers the a complete holistic pipeline for capturing, combining, and curating sequence data from GenBank. mtDNAcombine not only make the process easier and faster, undertaking all of the necessary processing steps within in one programme, R, but it also directs the user around idiosyncratic issues associated with data compilation from GenBank that might otherwise have been over looked. Issues around nomenclature, for example, are rarely considered but can significantly reduce the number of available samples found for a species/gene when performing an automated search. Equally issues around the style of data deposition, single representative haplotypes vs individual samples, can have a significant impact of the validity of downstream analysis if not dealt with. By programmatically handling these factors mtDNAcombine both removes the chance of sources of complications going unnoticed and ensures the user consciously handles potentially problematic datasets.

Whilst mtDNAcombine has been specifically designed to tackle the challenges associated with sourcing and aggregating data from multiple studies, complexities do remain. For example, although mtDNAcombine automatically handles the presence of duplicated samples resulting from RefSeq data, repeated entries for a single sample can also arise in datasets compiled from multiple sources due to re-uploaded/re-sequenced samples. This occurs most frequently when multiple studies sample a single museum specimen. Re-sequenced samples are often hard to identify, and recognising repeated use of published alternative ID numbers (such as specimen numbers) are sometimes the only indications that the same individual has been sequenced by multiple studies. Unfortunately, there is no simple programmatic way to identify re-sequenced samples given the information provided in GenBank. It should be noted that the occasional duplicate entry in a moderate sample size would be unlikely to cause a significant skew in any recovered population history. Despite this, users compiling large genetic datasets from multiple studies should be conscious that this source of duplicate entry exists and needs to be avoided.

At the moment, the lack of standardisation in the data upload process is another factor that exacerbates the inevitable complexities of combining data from multiple origins. Whilst some samples, sequenced early in the molecular era, are allowably poorly documented, we urge people to be careful when uploading data today. The more meta-information about a sample that is included online, alongside sequence data, the more likely that sequence will be usable by others. Equally, with the volume of data available today, the accuracy of associated meta-data and sequence tags/labels is vital for ensuring the data are retrievable when large scale, automated, searches are used. Many sequences are ‘lost’ due to inaccuracies or inconsistencies in how the data are uploaded to repositories, rendering a proportion of the potential data unusable.

We suggest that a focus on quality control for additional information about each sample will make a noticeable difference to the ease with which public databases can be mined for relevant information and this exceptional resource exploited. Where accompanying information is not uploaded to repositories, we urge authors to make this information easily accessible to readers. For example, downstream use will be facilitated by providing haplotype frequency data or detailed sampling location data as supplementary files (ideally well formatted text files which are easy to process) rather than embedded tables or images within manuscripts. We hope that our discussion, whilst highlighting common pitfalls, provides solutions and suggestions to guide the process of compiling data sets from online databases.

Although covered in several recent reviews [29, 52], whilst BSPs are a powerful tool for demographic reconstruction, over-interpretation continues to be an issue and hence its dangers are worth re-iterating. Unsurprisingly, problems are greatest with weaker data: smaller sample sizes, uneven sampling strategy, and/or when drawn from a species with strong population substructure [29, 30]. Despite using filtering criteria appropriate for a BSP analysis throughout the mtDNAcombine pipeline it is still critical that BSP outputs are reviewed cautiously and with appropriate consideration for the biological likelihood and relevance of any result [53]. In our illustrative BSPs we have one species, the purple sandpiper (Fig. 4a), that produced a flat profile. Whilst this may represent a true population history, it could also be indicative of a limitation of the BSP method given the data provided [52]. Interpretation of BSP plots must always be done with appropriate consideration for key factors such as the data quality, data quantity, and species life history.

## Conclusions

As the amount of genetic data stored in public databases like GenBank grows so the potential for building large datasets from multiple studies also grows. Indeed, utilising publicly available data in this way can offer the opportunity to explore new and exciting avenues of research. Yet, compiling multi-study data is challenging, with many complexities. The R package presented here is designed to provide a streamlined pipeline that guides the user through the process of assembling and handling multi-study sequence data from GenBank. We hope that this set of tools will help make the process of compiling and formatting large data sets more accessible for researchers in the future.

### Availability and requirements

The mtDNAcombine source code and full vignette, including installation instructions, can be found at the project home page: https://github.com/EvolEcolGroup/mtDNAcombine.

mtDNAcombine runs on Linux or MS-Windows operating systems and requires the software environment R (version ≥ 3.4), freely available from CRAN at https://cran.r-project.org/.

It is distributed under license GNU GPL (≥ 2).

There are no restrictions to use by non-academics.

## Supplementary Information

**Additional file 1.** Haplotype network diagrams from the white wagtail dataset. On the left is the initial dataset, including a sample that was classed as an extreme outlier (in this study, a single sample that was separated from all others by >30 base changes). On the right, the same dataset after removal of the single extreme outlier.

**Additional file 2.** Input files needed to recreate the plots in this paper: Tracer output files for three species.

**Additional file 3.** Input files needed to recreate the plots in this paper: raw sequence data for alignment.

**Additional file 4.** Code to create the plots in this paper presented as a R markdown file.

## Data Availability

mtDNAcombine source code and full vignette can be found at: https://github.com/EvolEcolGroup/mtDNAcombine. A sample dataset is available within the R package. The data used as examples in this paper were derived from publicly available data in GenBank (https://www.ncbi.nlm.nih.gov/genbank/); accession numbers are as follows, AF407040.1, AF526472.1, AF526476.1, AF526478.1, AF526486.1, AF526496.1, AF526498.1, AH011269.2–AH011297.2, AY156131.1–AY156134.1, AY681496.1–AY681524.1,, AY681533.1–AY681562.1, AY681568.1, AY681571.1–AY681591.1, AY681595.1–AY681699.1, AY681702.1, AY681711.1, AY681728.1, AY681729.1, AY703261.1–AY703446.1, DQ188164.1, EU166960.1, FJ547513.1–FJ547517.1, GQ870799.1–GQ870867.1, GU816849.1, JN715428.1, JN715468.1, KC759308.1, KC969098.1, KF194129.1, KF194140.1, KF194157.1, KJ455349.1, KJ455507.1, KM078781.1, KP765391.1–KP765402.1, KT031286.1, KT031292.1, KT031305.1–KT031326.1, KT031331.1, KT736087.1, NC_025609.1, NC_029229.1. Raw input files (Additional Files 2-3), and code (Addittional File 4) needed to create the plots in this paper are also provided.

## Abbreviations

mtDNA: Mitochondrial DNA
LGM: Last Glacial Maximum
BSPs: Bayesian skyline plots
WGS: Whole genome sequencing
PSMC: Pairwise sequentially Markovian coalescent method
MSMC: Multiple sequential Markovian coalescent method
*N*_e_: Effective population size
MJN: Median joining network
