# Supplementary figures and images for: mtDNAcombine: tools to combine sequences from multiple studies
Source: BMC Bioinformatics. 2021 Mar 9;22:115. doi: 10.1186/s12859-021-04048-0 (PMC7945669; doi:10.1186/s12859-021-04048-0)

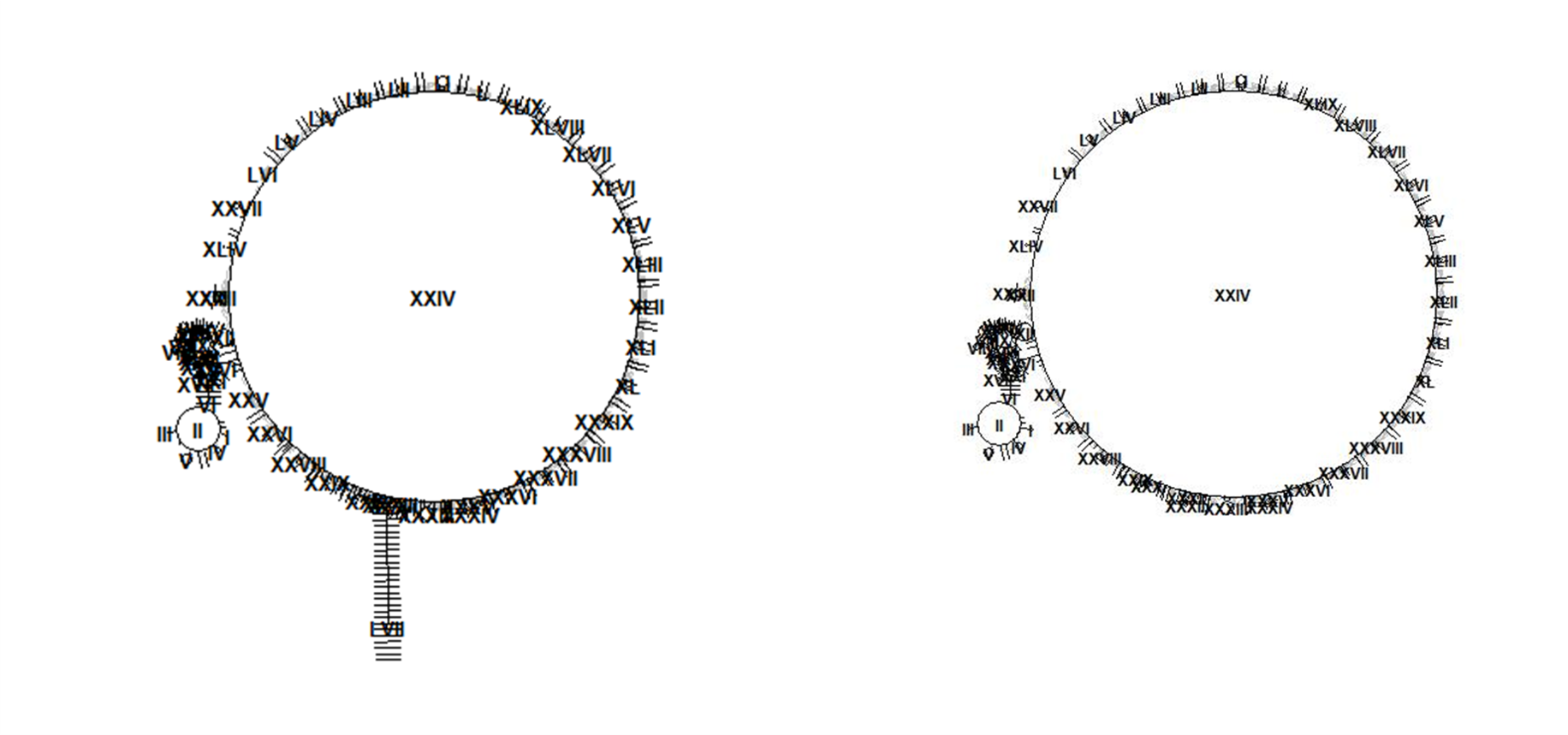

Supplement: Supplementary file 1 — Additional file 1. Haplotype network diagrams from the white wagtail dataset. On the left is the initial dataset, including a sample that was classed as an extreme outlier (in this study, a single sample that was separated from all others by >30 base changes). On the right, the same dataset after removal of the single extreme outlier. [file 12859_2021_4048_MOESM1_ESM.png]
